# Optimal processing of surface facial EMG to identify emotional expressions: A data-driven approach

**DOI:** 10.3758/s13428-024-02421-4

**Published:** 2024-05-21

**Authors:** J. M. Rutkowska, T. Ghilardi, S. V. Vacaru, J. E. van Schaik, M. Meyer, S. Hunnius, R. Oostenveld

**Affiliations:** 1 Donders Institute for Brain, Cognition and Behaviour, Radboud University, Nijmegen, The Netherlands; 2 Department of Psychology, University of Zurich, Zurich, Switzerland; 3 Jacobs Center for Productive Youth Development, University of Zurich, Zurich, Switzerland; 4 Centre for Brain and Cognitive Development, Department of Psychological Sciences, Birkbeck, University of London, London, UK; 5 Vrije Universiteit Amsterdam, Amsterdam, the Netherlands; 6 Department of Psychology, New York University - Abu Dhabi, Abu Dhabi, United Arab Emirates; 7 Behavioral Science Institute, Radboud University, Nijmegen, The Netherlands; 8 NatMEG, Karolinska Institutet, Stockholm, Sweden

**Keywords:** Facial electromyography, Surface electromyography, Emotion, Optimal pipeline, Multiverse

## Abstract

Surface facial electromyography (EMG) is commonly used to detect emotions from subtle facial expressions. Although there are established procedures for collecting EMG data and some aspects of their processing, there is little agreement among researchers about the optimal way to process the EMG signal, so that the study-unrelated variability (noise) is removed, and the emotion-related variability is best detected. The aim of the current paper was to establish an optimal processing pipeline for EMG data for identifying emotional expressions in facial muscles. We identified the most common processing steps from existing literature and created 72 processing pipelines that represented all the different processing choices. We applied these pipelines to a previously published dataset from a facial mimicry experiment, where 100 adult participants observed happy and sad facial expressions, whilst the activity of their facial muscles, *zygomaticus major* and *corrugator supercilii*, was recorded with EMG. We used a resampling approach and subsets of the original data to investigate the effect and robustness of different processing choices on the performance of a logistic regression model that predicted the mimicked emotion (happy/sad) from the EMG signal. In addition, we used a random forest model to identify the most important processing steps for the sensitivity of the logistic regression model. Three processing steps were found to be most impactful: baseline correction, standardisation within muscles, and standardisation within subjects. The chosen feature of interest and the signal averaging had little influence on the sensitivity to the effect. We recommend an optimal processing pipeline, share our code and data, and provide a step-by-step walkthrough for researchers.

**Supplementary Information:**

The online version contains supplementary material available at 10.3758/s13428-024-02421-4.

## Introduction

Surface facial electromyography (EMG) is commonly used in the affective science and psychological fields as a non-invasive tool to assess subtle facial emotional expressions in order to study emotional cognition and facial mimicry (e.g. Kret et al., 2013a). Electrodes placed on the skin record the signal from facial muscles that represents the magnitude and the frequency of the action potentials responsible for the muscles’ contraction when expressing an emotion. Importantly, before the EMG signal can be analysed, it needs to go through different processing steps which require researchers to make a series of decisions. Crucially, they must choose which feature extracted from the data best summarises the facial muscle activity, and which standardisation method best deals with the between-participants and between-muscle variance that is unrelated to emotional expressions and the studied effect.

To identify the most commonly used processing steps in the literature, we conducted a literature review prior to this study that included 31 papers on emotional facial mimicry published between 2007 and 2020. We identified a variety of processing practices employed in existing literature on adults and children, with over 15 unique combinations of extracting features of interest and standardisation methods. In addition, we also observed that in many cases, some processing details were unclear or omitted. For new research, it is inefficient to systematically evaluate many different analysis pipelines on one’s data, especially given the risk of this resulting in selective reporting and p-hacking (Wicherts et al., 2016). However, to date, there has been no systematic investigation on how the multitude of choices in the analysis pipeline influence the quantification of the EMG signal in retaining the emotion-related information, and consequently, to what extent it can be used to examine facial emotional expressions in research. This paper aims to establish an optimal standard for processing facial EMG data. To this end, we outline how emotional expressions are measured using facial EMG, review the most common approaches for preprocessing, quantifying, and analysing EMG features, and review which standardisation methods are used to reduce within- and between-subject variance. Subsequently, using a large existing facial EMG dataset with an established emotional contrast effect (Vacaru et al., 2021), we systematically compare processing methods and report on the processing decisions that retain the maximum emotional information, while addressing the extrinsic, unwanted variability in the EMG signal.

### Quantifying emotional expressions using facial EMG

Surface facial EMG is a widely implemented method in research on emotions with adult (e.g., Fridlund & Cacioppo, 1986; van Boxtel, 2010; Kret et al., 2013a) and developmental populations (e.g., Addabbo et al., 2020; Kaiser et al., 2017; Schröer et al., 2022). Pioneering work by Cacioppo and colleagues (1986) and Larson and colleagues (2003) demonstrated that electromyographic activity of the facial muscles differentiates the emotional valence and intensity of an observed facial expression. In addition to the behavioural work of Ekman (1989) who described the facial action units characterising specific overt emotional facial expressions, the introduction of facial EMG advanced the emotion and affect information processing field by assessing also covert emotional processes. Facial EMG captures the activity of muscle action potentials, even when muscle contraction and movement is too small to be visible to the bare eye. That is why it has been adopted as a standard measure for detecting facial emotional expressions and their mimicry, that is, the mirroring of another person’s facial expression occurring outside one’s awareness (Fischer & Hess, 2017; Geangu et al., 2016; Vacaru et al., 2019).

By comparing the mean amplitudes of EMG signals from facial muscles related to specific emotions within a certain time interval, evidence has accumulated for its potential of reliably assessing several basic facial emotional expressions with EMG (e.g., happy, sad, angry, pain, surprise; Fischer & Hess, 2017; Seibt et al., 2015; Vacaru et al., 2019). For example, a happy expression is characterised by higher amplitudes in the *zygomaticus major* (ZM), a muscle involved in smiling, and lower amplitudes in the *corrugator supercilii* (CS), a muscle involved in frowning, compared to a resting state (Cacioppo et al., 1986; van Boxtel, 2010). The opposite pattern holds true for a sad expression. Due to the rapid advancement and relative “ease of use” of surface facial EMG, many fields of study complemented their methods with such recordings, even in the absence of prior electrophysiology expertise. While this allows researchers to bridge previously separated scientific fields or address new research questions, it also poses limits to the thorough understanding and appropriate execution of signal processing and data analysis. To our understanding, while there is wide agreement over the recording procedures (Cacioppo et al., 1986), there is no consensus on EMG signal processing. This is an important issue because the standardised electrode placement cannot account for the anatomical differences between participants’ faces and their facial muscles. Optimal signal processing can take these differences into consideration, whilst simultaneously capturing the variability in the EMG signal related to the research question (Halaki & Ginn, 2012).

### EMG signal preprocessing

An essential first step in the EMG signal analysis is preprocessing of the data, as it removes noise from the data and capitalises on the signal of interest. Any contribution to the recorded signal that did not originate from the muscle being studied can be considered noise, such as artefacts due to the electrodes moving relative to the skin, or the noise generated by electrical equipment (Kale & Dudul, 2009). The EMG signal is routinely filtered with a 20–500 Hz bandpass filter to encompass the optimal bandwidth for facial EMG (van Boxtel, 2001; 2010), although there might be slight differences in filter frequencies chosen by individual researchers that focus on different facial muscles (van Boxtel, 2001). In addition, a 50 Hz or 60 Hz notch filter is often employed to remove power-line interference (Altimari et al., 2012; van Boxtel, 2010). Data segments of relevance for further analysis (also called epochs) are then selected for further processing; these for instance correspond to experimental trials. A next step is to identify and remove segments affected by motion artefacts. The data are then full-wave-rectified, that is, negative values are converted to positive ones (Altmari et al., 2012). Subsequently, to smooth the data, the high-frequency rectified EMG signal is often passed through a low-pass filter (van Boxtel, 2010; Moody & McIntosh, 2011; de Klerk et al., 2018). For more information on the preprocessing of the EMG signal, see for example van Boxtel (2010), Vigotsky et al. (2018), Altimari et al. (2012), and Hamedi (2011). There appears to be little disagreement within the field on these individual steps in preprocessing surface EMG signals, hence their signal-analytical rationale and optimal settings are not further covered here. We instead focus on the subsequent quantification and normalisation of the EMG measure to compare muscles and conditions to detect emotional expressions.

### Quantifying and analysing EMG features

Following preprocessing, we still have a continuous signal consisting of many data points within each trial, that is, the EMG signal has a high temporal dimension. The next step is to reduce our signal to the temporal dimension of one trial, so to summarise all the data points within a trial with just one data point. Therefore, we need to find an index that best represents the signal in one trial by extracting from it the feature of interest. Our literature review identified three most commonly used features of interest: mean absolute value (MAV; e.g., Kret et al., 2013a, 2013b), root mean square (RMS; e.g., Datyner et al., 2017), and integrated EMG (iEMG; e.g., Minio-Paluello et al., 2020; for mathematical definitions of these features, see Phinyomark et al., 2012).

The most frequently used metric appears to be the MAV, an average of the absolute (full-wave-rectified) value of the EMG amplitude over the experimental time window of interest (i.e., the trial; Phinyomark et al., 2012). This is also sometimes referred to as average rectified value, average absolute value, or mean rectified value (Phinyomark et al., 2012; Clancy et al., 2002). A less frequently used feature of interest is the iEMG. It is the integral (area under the curve) of the rectified EMG signal; its values are often log_10_-transformed to reduce the impact of outliers (Moody et al., 2007). From a mathematical point of view, MAV and iEMG provide corresponding results, which means that after extracting MAV and iEMG from the same trial, the exact values will differ, but by a specific factor. Thus, the pattern of results, such as which value is higher and which is lower, will be the same. We have still decided to include both MAV and iEMG in our investigation, as it might make it easier for the researchers to compare their processing pipelines with ours. The least frequently used feature of interest is RMS (root mean square). It is calculated as the square root of the average (over the time window of interest) of the squared EMG amplitudes. There is evidence that both RMS and MAV are appropriate for estimating EMG amplitudes, but that RMS is more accurate when contraction level is high (i.e., higher than 10% of maximum voluntary contraction of the muscle), and MAV when it is low (Clancy et al., 2002). Facial mimicry research is mostly concerned with subtle changes in the activation of facial muscles, which suggests that MAV could be a better feature of interest than RMS. The influence of the choice between MAV, RMS, and iEMG on the detectability of mimicked emotional expressions is investigated in this paper, alongside the effect of standardisation practices.

### Dealing with within- and between-subjects variance

The third step in the analysis of the EMG signal is standardisation (often referred to as normalisation). The EMG signal varies within subjects due to the physiological and anatomical differences between muscles. Furthermore, the EMG signal varies between subjects due to differences in the anatomy of the same muscle, different placement of electrodes (Besomi et al., 2020; van Boxtel, 2010), and different facial expressions and levels of emotional mimicry. The purpose of standardisation is to enable comparisons of task-induced experimental effects between muscles and between individuals. We have identified three standardisation methods typically used in the literature that examines facial EMG: baseline correction, standardisation within muscles, and standardisation within subjects. The first method is baseline correction, and it is done by expressing the EMG amplitude during the experimental time window of interest as a proportion of the baseline activity (**baseline division**; e.g., Kret et al., 2013b), or subtracting the baseline from it (**baseline subtraction**; e.g., Drimalla et al., 2019). The baseline is usually a time window before the experimental time window of interest, when no emotional stimuli are presented. Although we found both types of baseline correction frequently used in the literature, baseline division has been proposed to be more appropriate than baseline subtraction (van Boxtel, 2010). This is because the EMG signal recorded from facial muscles, unlike other types of psychophysiological responses, is measured on a ratio scale (having absolute zero origin), rather than an interval scale (not having a zero origin). The second standardisation method is **standardisation within muscles**, and it involves expressing the EMG signal amplitude as *z*-scores over each muscle of each participant. It is often used in combination with baseline correction, and sometimes instead of baseline correction, in studies with small infants, when their baseline activity is contaminated and cannot be reliably determined (e.g. de Klerk et al., 2019). The third standardisation method is **standardisation within subjects**, which involves expressing the EMG signal amplitude as *z*-scores over all the muscles of each participant (e.g. de Klerk et al., 2018, 2019). From the literature review, it is not entirely clear how often this type of standardisation is employed, due to often vague descriptions of the processing steps. This method of standardisation might only be useful when comparing responses of a specific muscle within a specific person.

### Current study

The aim of the current study was to establish optimal processing practices for surface facial EMG data in emotional and facial mimicry research. As the field of psychological research on emotional expressions and facial mimicry conducted with facial EMG is still developing, different processing practices of the facial EMG signal are currently being used, but the rationale behind employing specific practices is not always clear. EMG research with human participants is a costly and time-consuming process, and it is especially challenging with children and infants due to the restrictions in instructing the participants, resulting in only a few useful trials and many motion artefacts. Therefore, it is important to identify the methods that optimise the quantification of the EMG signal to be sensitive for the detection of emotion effects. Importantly, it involves not only detecting the main effects of emotional expressions on the EMG signal, but also being able to detect task-specific individual differences and interactions that might be small.

In this paper, we took a data-driven approach examining the effects of the above-mentioned, commonly chosen features of interest (MAV, RMS, iEMG) and standardisation methods on previously collected adult facial EMG data from a facial mimicry experiment (Vacaru et al., 2021). In addition, our literature review highlighted another processing step of the EMG signal, signal averaging, where the trials from one muscle in each condition are averaged together. This can be done before or after other processing steps (sometimes referred to as data reduction). We created 72 individual processing pipelines from the different combinations that result from systematically varying all the possible choices in processing steps: signal averaging and data reduction, feature of interest, baseline correction, standardisation within muscle, and standardisation within subject. We resampled the data from 100 participants by splitting it into three sub-samples of 33 participants, a sample size that is representative of the average sample size in the literature. We repeated this 500 times, resulting in 1500 sub-samples that we used for the analysis. This enabled us to repeatedly evaluate the performance of each processing pipeline independently of the distribution of participants between the samples. To assess the extent to which the EMG signal can be used to detect a mimicked emotion (happy or sad), we fitted a logistic regression model to the data for each sub-sample processed with each pipeline. We averaged the performance of the models for each pipeline across different sub-samples to evaluate which processing pipeline leads to the best detectability of mimicked emotion from the EMG signal. We then used a random forest model to quantify which processing steps in the pipelines had the biggest impact on the detectability of mimicked emotion. Following these analyses, we made recommendations for the optimal processing choices for the EMG data in emotional, facial mimicry research. Additionally, we provide a walkthrough for a recommended pipeline. All data and the scripts used in the paper are available online in a data repository (Rutkowska et al., 2023) and on GitHub (https://github.com/TommasoGhilardi/EMG_Pipelines).

## Methods

### Data acquisition

**Fig. 1:**
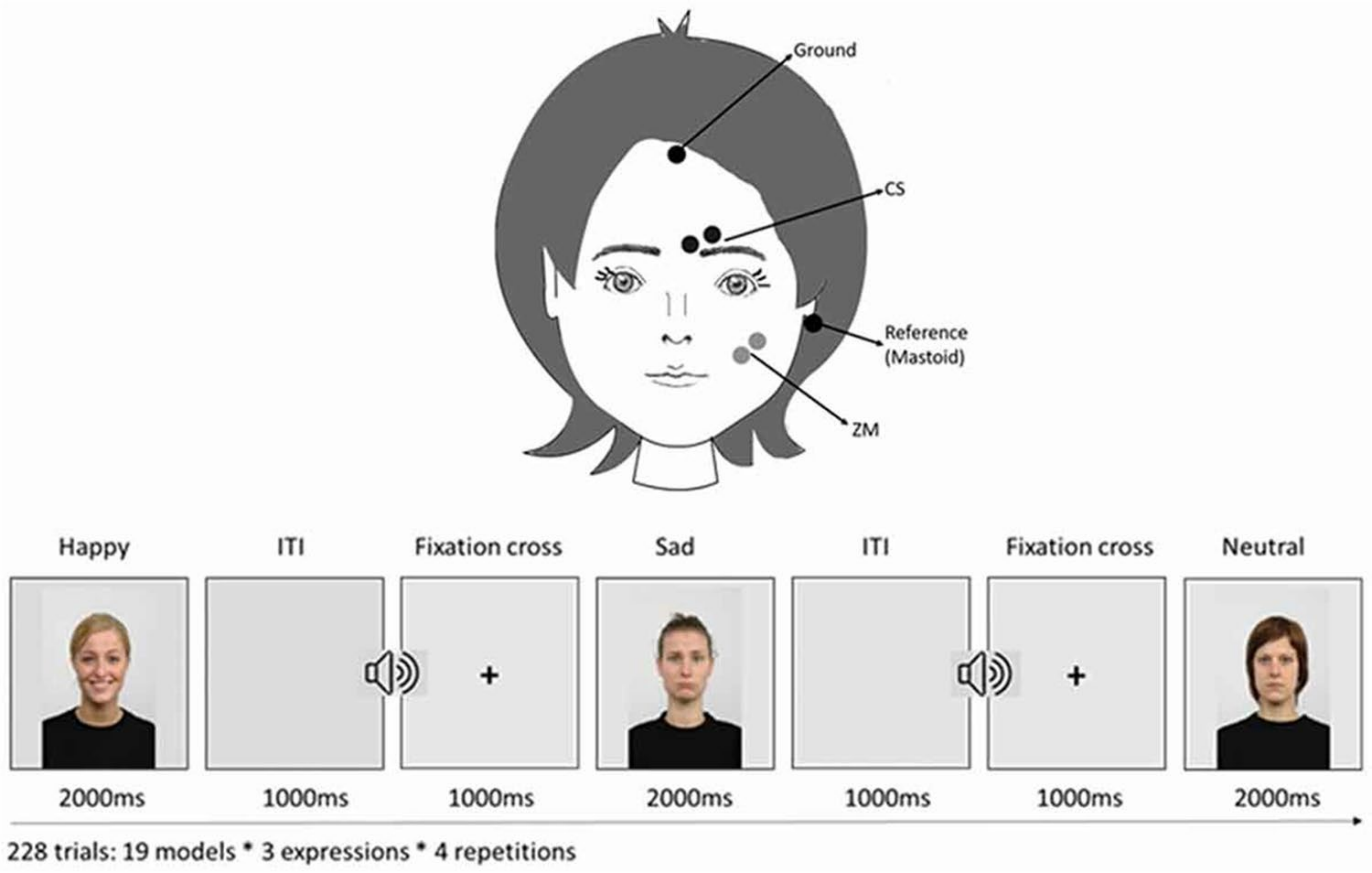
Schematic illustration of the study design and the positions of the electrodes assessing the activation over the ZM and CS facial muscles. Taken from Vacaru et al. (2021)

The data used in this project was collected by Vacaru and colleagues (Vacaru et al., 2021) to study the modulation of emotional facial mimicry by attachment tendencies in healthy adults. Facial surface EMG recordings were collected from 100 participants (68 females; *M*_age_ = 24.54 years, *SD*_*a*ge_ = 3.90, range: 18–35) recruited in a middle-sized city in the Netherlands. The signal was recorded from two muscles—ZM and CS—used to assess emotional mimicry from happy and sad emotional expressions, respectively. EMG responses were measured via 4-mm Ambu-Neuroline 700 Ag/AgCl surface electrodes, using Brain Vision Recorder (Brainproducts GmbH, 2009). The participants’ skin was first cleaned using a scrubbing gel (Nuprep Skin Prep Gel) and medical alcohol. Next, the electrodes were applied with a bipolar montage and 10 mm inter-electrode distance between their centres on the muscle sites of interest, and two additional areas for the ground electrodes on the forehead and a common reference electrode on the mastoid bone behind the ear (see Fig. 1). Some conductive OneStep Cleargel was added to the already pre-gelled electrodes to improve impedances. Impedances were kept below 10 kilohms. A sampling rate of 2500 Hz was used with a high-pass cutoff frequency of 10 Hz and low-pass cutoff frequency of 1000 Hz.

The participants watched stills of emotional facial expressions of white female models (Radboud Faces Database; Langner et al., 2010). In the original study, happy, sad, and neutral facial expressions were used, but this paper uses the data from the happy and sad expressions only because there is no established effect of neutral expressions on facial muscles. Nineteen models featured happy and sad facial expressions, each repeated four times, for a total of 152 trials, presented in a pseudo-randomized manner (MIX; van Casteren & Davis, 2006). Each trial lasted 4000 ms: 1000 ms fixation cross, 2000 ms stimulus presentation, and 1000 ms inter-stimulus interval (see Fig. 1). With the onset of the fixation cross, a short beep was played as an attention getter, after which the stimulus was displayed on a computer monitor.

### Preprocessing

Raw data files acquired from Vacaru and colleagues (Vacaru et al., 2021) were preprocessed with a custom MATLAB script based on the FieldTrip toolbox (Oostenveld et al., 2010). To obtain bipolar signals, the signal from one electrode on each muscle site (ZM and CS) was re-referenced to the other electrode from the same muscle site. Next, a 20–500 Hz bandpass filter was applied. The mean and standard deviation (SD) were calculated for the rectified data in each channel for each participant. For artefact rejection, the data were divided into 1000-ms-long epochs. Epochs with mean amplitude above or below three SD from the grand mean in at least one channel were identified and flagged for rejection. Next, the data were re-divided into trials starting 500 ms before the stimulus onset (baseline) and ending 2000 ms after the stimulus onset. Trials overlapping with the flagged artefacts were excluded from the analysis (M = 0.42% trials, maximally five trials per participant).

### Creating different processing pipelines

We conducted a literature review to find the most frequently used methods for quantifying and analysing EMG features and for dealing with within- and between-subject variance (see Introduction). The starting point for the review consisted of domain-specific articles the authors were already familiar with, and the others were found through those article’s references and from reverse referencing. Forty-seven papers that used surface facial EMG to measure facial mimicry or emotion matching in adults and children were found (see Article list in Supplementary materials). From these articles, six consecutive processing steps were identified:

1. Signal averaging:

- None: the step was skipped, and the raw signal was used.
- Average: the data were averaged within one participant across trials for each muscle for each condition before further processing.

2. Feature of interest:

- RMS: root mean square was extracted from each trial.
- MAV: mean absolute value was extracted from each trial.
- iEMG: integral (area under the curve) was extracted from each trial.

3. Baseline correction:

- None: the step was skipped.
- Divide by baseline: the signal from a trial was divided by the mean signal from the baseline.
- Subtract the baseline: the mean signal from a trial’s baseline was subtracted.

4. Standardization within muscle:

- None: this step was skipped.
- *Z*-score: a *z*-score was calculated over each muscle within participants.

5. Standardization within subject:

- None: this step was skipped.
- *Z*-score: a *z*-score was calculated over all the muscles within participants.

6. Data reduction:

- None: this step was skipped.
- Average: the data were averaged within one participant across trials for each muscle for each condition.

**Fig. 2:**
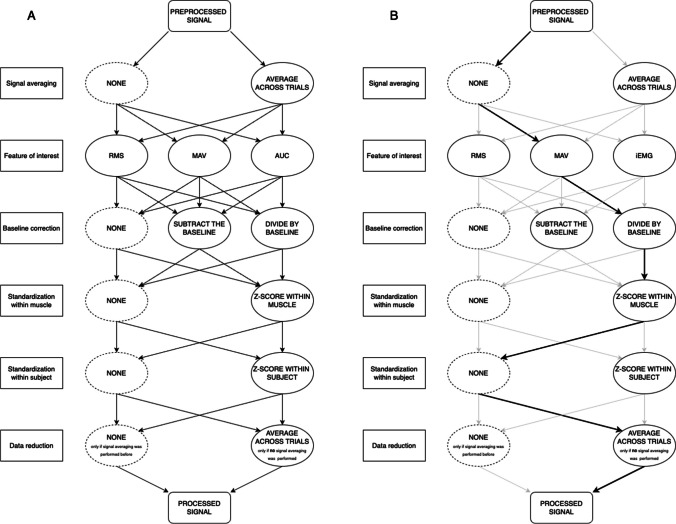
**A** A diagram of processing steps and their possible sequences. All pipelines included a data averaging step, either during signal averaging (first step) or during data reduction (last step), but the data were never averaged twice. **B** An example pipeline, including (1) no signal averaging in the first step, (2) mean absolute value as a feature of interest, (3) division by baseline as a baseline correction, (4) *z*-scoring within each muscle within participants, (5) no *z*-scoring between muscles within participants, and (6) averaging across trials in the data reduction step. It corresponds to pipeline Aa_MAV_Bd_Ms_Sn (see Table 1 in Supplementary materials and *Naming the processing pipelines*)

Seventy-two different processing pipelines were created based on these steps (see Fig. 2 and Table 1 in Supplementary materials) in MATLAB using the Fieldtrip toolbox (Oostenveld et al., 2010). Importantly, all pipelines included the same data averaging step, where the data were averaged within one participant across trials for each muscle for each condition, either during signal averaging (1b) or during data reduction (6b), but the data were never averaged twice.

### Naming the processing pipelines

We used a consistent naming scheme for the pipelines based on the processing steps that they entail. Every pipeline was named accordingly to the following template: Ax_xxx_Bx_Mx_Sx, reflecting every processing step (**A**veraging, **B**aseline correction, standardisation within **M**uscle, standardisation within **S**ubject), with the processing choice to be filled (x). All the pipeline names and explanations can be found in Table 1 in the Supplementary materials.

1. The first two letters refer to whether the data were averaged across trials at the beginning or at the end of the processing (whether step 1 or step 6 in Fig. 2 was carried out): ‘**A**’ for ‘Averaged’, and ‘**b**’ for **b**efore, or ‘**a**’ for **a**fter; Thus, ‘**Ab**’ stands for ‘averaged before’ step 1 was carried out, and ‘**Aa**’ stands for ‘averaged after’ step 6 was carried out.
2. The following three or four letters refer to the feature of interest used (step 2 in Fig. 2): ‘**iEMG**’ for **i**ntegral of the **EMG**, ‘**RMS**’ for **R**oot-**M**ean-**S**quare, and ‘**MAV**’ for **M**ean **A**bsolute** V**alue.
3. The following two letters refer to the baseline correction used (step 3 in Fig. 2): ‘**B**’ for **B**aseline, and ‘**s**’ for **s**ubtraction, or ‘**d**’ for **d**ivision, or ‘**n**’ for **n**o correction; Thus, ‘**Bs**’ stands for baseline subtraction, ‘**Bd**’ stands for baseline division, and ‘**Bn**’ stands for no baseline correction.
4. The following two letters refer to whether the standardisation within muscle was used (step 4 in Fig. 2): ‘**M**’ for within **M**uscle, and ‘**s**’ for **s**tandardised or ‘**n**’ for **n**ot standardised; Thus, ‘**Ms**’ stands for standardised within muscles, and ‘**Mn**’ stands for not standardised within muscle.
5. The last two letters refer to whether the standardisation within subject was used (step 5 in Fig. 2): ‘**S**’ for within **S**ubject, and ‘**s**’ for **s**tandardised or ‘**n**’ for **n**ot standardised; Thus, ‘**Ss**’ stands for standardised within subject, and ‘**Sn**’ stands for not standardised within subject.

As an example, let us take the pipeline from Fig. 2B.

1. The signal was averaged after the other processing steps (in step 6): ‘**Aa**’.
2. The feature of interest used was mean absolute value: ‘**MAV**’.
3. The baseline correction method was baseline division: ‘**Bd**’.
4. The standardisation within muscle was carried out: ‘**Ms**’.
5. There was no standardisation within subject: ‘**Sn**’.

Thus, the pipeline name is: Aa_MAV_Bd_Ms_Sn.

### Resampling

We used resampling on the large dataset to evaluate the pipeline performance across different distributions of data, making our results more robust, whilst using a sample size that is representative of the usual sample sizes in the field. The data were first exported to RStudio (version 2023.06.1, RStudio Team, 2020). Then, the data from the 100 participants were randomly resampled without replacement 500 times into three sub-samples of 33 participants. We chose a sub-sample size of 33 based on the median number of the sample sizes used in the studies included in the literature review (median = 34). Furthermore, we decided to make the subsample size 33 instead of 34, so that with each resampling we were able to make three non-overlapping subsamples instead of two. Resampling the data 500 times into three sub-samples resulted in a final number of 1500 sub-samples for the analysis.

### Evaluating pipeline performance with logistic models

Each of the 1500 sub-samples of the data was processed with each of the 72 pipelines. Before any statistical analysis, a final artefact rejection was conducted on the data of each sub-sampled pipeline. Data exceeding two standard deviations from the mean was considered an artefact and rejected. After cleaning the data, we fitted a logistic model to each of the sub-sampled pipelines, estimated with maximum likelihood. A logistic model is a statistical model that is used for predicting binary outcomes (i.e., emotion: happy and sad). The model uses a logistic function (also called a sigmoid function) to model the probability (between 0 and 1) that an observation belongs to a certain class. With the logistic model being applied to each of the pipelines and each of the 1500 sub-samples, this comprises a multiverse analysis that enables us to systematically explore the impact of different processing pipelines on the EMG data’s ability to predict the mimicked emotion (Steegen et al., 2016; Harder, 2020) and to identify the pipeline features with the best results.

All logistic models were fitted with emotion as the dependent variable (happy and sad). The electrophysiological data extracted from ZS and CS muscles and their interactions were added as independent variables (Emotion ~ CS * ZS). After fitting the models, we calculated the sensitivity (true positive rate) and specificity (false positive rate) for each of them using the performance_roc function from the performance package (Lüdecke et al., 2021), and then determined the area under the curve for each model using the area_under_curve function from the BayestestR library (Makowski et al., 2019).

After fitting all models, one area under the curve (AUC) value was calculated for each pipeline by averaging over sub-samples. The AUC is a commonly used metric for evaluating the performance of binary classification models, including logistic regression models (Bradley, 1997). The AUC provides a single scalar value that represents the overall performance of a model by summarising the model's ability to distinguish between the rates of true positives (sensitivity) and false positives (specificity). AUC ranges in value from 0 to 1, with a value of 0.5 indicating a model that performs no better than chance and a value of 1 indicating a model that perfectly separates the two classes. Thus, the higher the AUC, the better the logistic model is at classifying the mimicked emotion based on the EMG data.

### Evaluating different processing choices with a random forest

To further investigate which preprocessing steps had the strongest impact on the results of logistic models, a random forest analysis using the randomForest package was conducted (Fife & D’Onofrio, 2022, version 4.7-1.1). This machine learning algorithm creates multiple decision trees that predict the outcome variable, making it a useful tool for determining which variables had the most substantial impact on the prediction. In our case, we used a random forest to determine which processing step had the biggest impact on the ability to determine the mimicked emotion from the EMG signal, measured by the AUC of the logistic models.

The AUC values from all the logistic models were split into a training and test dataset with an 80:20 ratio. Before running the model, the function tuneRF was used to determine the best mtry value, which determines the number of variables selected at each split. The random forest model was then fitted on the AUC values of the training dataset, with the predictors being the different processing choices: feature of interest (RMS, MAV, iEMG), signal averaging (before or after other processing steps), baseline correction (none, divide by baseline, subtract the baseline), standardisation within subjects (none or *z*-scores), and standardisation within muscle (none or *z*-scores). The model was run with a parameter of mtry of 2 for 1000 trees and showed convergence. To evaluate the model's robustness, the results were then fitted to the test dataset, and the root mean squared error (RMSE) was used to assess the model's goodness of fit. This analysis helped to identify which preprocessing steps had the strongest impact on the AUC of the logistic model, reflecting the detectability of emotions from the EMG signal preprocessed by each pipeline. We have also generated partial dependence plots showing predicted AUC for each level of each variable in our random forest model. These values reflect how each processing choice, such as choosing to standardise within muscle or not, influences predicted detectability of emotions from the EMG signal.

## Results

### Pipeline performance

**Fig. 3:**
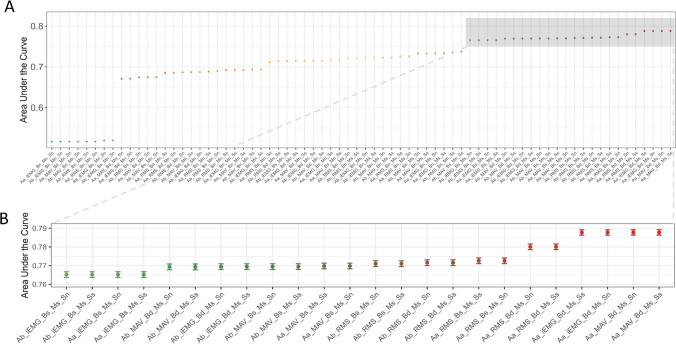
**A** The results of the analysis of the resampled data processed with different pipelines, with the logistic models predicting emotional expression (happy or sad). The area under the curve (AUC) represents the overall performance of the models, with higher AUC meaning better performance, and AUC > 0.5 indicating better performance than chance. The AUC is averaged over all 1500 subsamples of data, and standard deviation error bars are displayed for each pipeline. **B** The results for the top 24 performing pipelines (AUC > 0.75) are displayed.

The averaged area under the curve (AUC) for each pipeline is compared in Fig. 3. AUC values ranged from 0.52 to nearly 0.79. The following conclusions were drawn:

1. *The pipelines that include only extracting a feature of interest and signal averaging perform worse than other pipelines that include more processing steps.*

Those pipelines perform only slightly better than chance (AUC = 0.52) because they do not implement any baseline correction or standardisation, either within muscles or participants. That means that they do not account for the unwanted variability in the data that arises due to anatomical differences between muscles and people that can hinder the detection of emotional expressions from the EMG signal. These are for instance pipelines: Ab_iEMG_Bn_Mn_Sn, Aa_MAV_Bn_Mn_Sn, or Aa_RMS_Bn_Mn_Sn.

2. *Standardisation within muscle is important*.

Standardisation within muscle by *z*-scoring was present in all top-performing pipelines, that is, pipelines with AUC > 0.75, which shows that it is important independently of other processing choices. To see the importance of the standardisation within muscle, let us compare the pipelines with the same signal averaging and feature of interest: Aa_MAV_Bd_Ms_Ss (includes standardisation within muscles and subjects, and baseline correction by division; AUC = 0.79) and Aa_MAV_Bd_Mn_Ss (includes standardisation within subjects and baseline correction by division, but not standardisation within muscles; AUC = 0.71) or even Aa_MAV_Bn_Ms_Sn (includes only standardisation within muscles; AUC = 0.74).

3. *Different processing steps and choices interact with each other*.

The impact of some processing choices on the pipeline performance is sometimes dependent on other present processing choices.

- *Performing baseline correction (either by dividing by baseline or subtracting it) has a more positive impact if the pipeline includes standardisation within muscle.*

For instance, compare the pipeline with the same signal averaging and feature of interest, and no standardisation within subject: Aa_iEMG_Bd_Ms_Sn (includes both standardisation within muscle and baseline correction by division; AUC = 0.79) with Aa_iEMG_Bd_Mn_Sn (includes only baseline correction by division; AUC = 0.68) and Aa_iEMG_Bn_Ms_Sn (includes only standardisation within muscle; AUC = 0.74): The combination of baseline correction and standardisation within muscle yields the best result. All top-performing pipelines (with AUC > 0.75) include standardisation within muscle combined with a baseline correction step (either division by baseline or its subtraction).

b. *Standardisation within subject has little effect if the pipeline includes standardisation within muscle as well, but can be beneficial otherwise.*

For instance, compare the pipelines that differ only in the inclusion or exclusion of standardisation within subject: Aa_MAV_Bd_Ms_Sn and Aa_MAV_Bd_Ms_Ss, both AUC = 0.79, or Aa_RMS_Bs_Ms_Ss and Aa_RMS_Bs_Ms_Sn, both AUC = 0.77. In contrast, including standardisation within subject if there is no standardisation within muscle improves the pipeline performance. For instance, compare the pipelines that differ only in the inclusion or exclusion of standardisation within subject: Ab_iEMG_Bs_Mn_Ss (AUC = 0.71) and Ab_iEMG_Bs_Mn_Sn (AUC = 0.52), or Aa_MAV_Bs_Mn_Ss (AUC = 0.72) and Aa_MAV_Bs_Mn_Sn (AUC = 0.68).

4. *There is not one best feature of interest or signal averaging practice.*

We did not find systematic differences between the performance of the pipelines that include different features of interest (MAV, RMS, or iEMG) or different signal averaging practices (before or after other processing steps). Thus, those processing choices do not have a big impact on the ability to detect emotional expressions from the EMG signal and should be considered in combination with other processing steps.

### The impact of processing choice on the pipeline performance

**Fig. 4:**
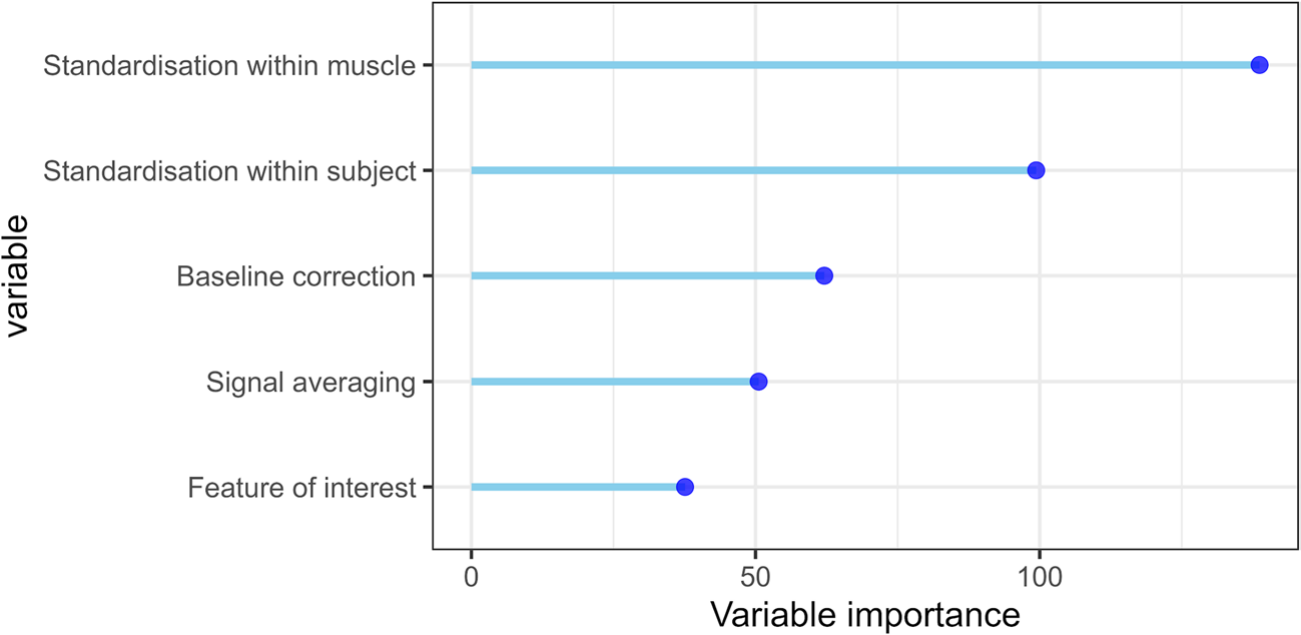
Random forest model variable importance, measured with mean decrease in accuracy, in predicting pipeline performance (measured with average AUC). The higher the variable importance, the more impact it had on the performance of the pipelines. *Note*: The signal averaging variable refers to the choice to average before or after other processing steps

Indicating the robustness of the random forest model, the RMSE of the test model showed a good fit, RMSE = 0.062. The importance of each variable choice is presented in Fig. 4 using the mean decrease in accuracy. This measure can be interpreted as the decrease in the accuracy of the model when the values of the variable are randomly shuffled, and other variables are kept intact. Thus, the more the model accuracy suffers when the variable is kept random, the more important the variable is for the ability to detect emotions from the EMG signal by the logistic models.

**Fig. 5:**
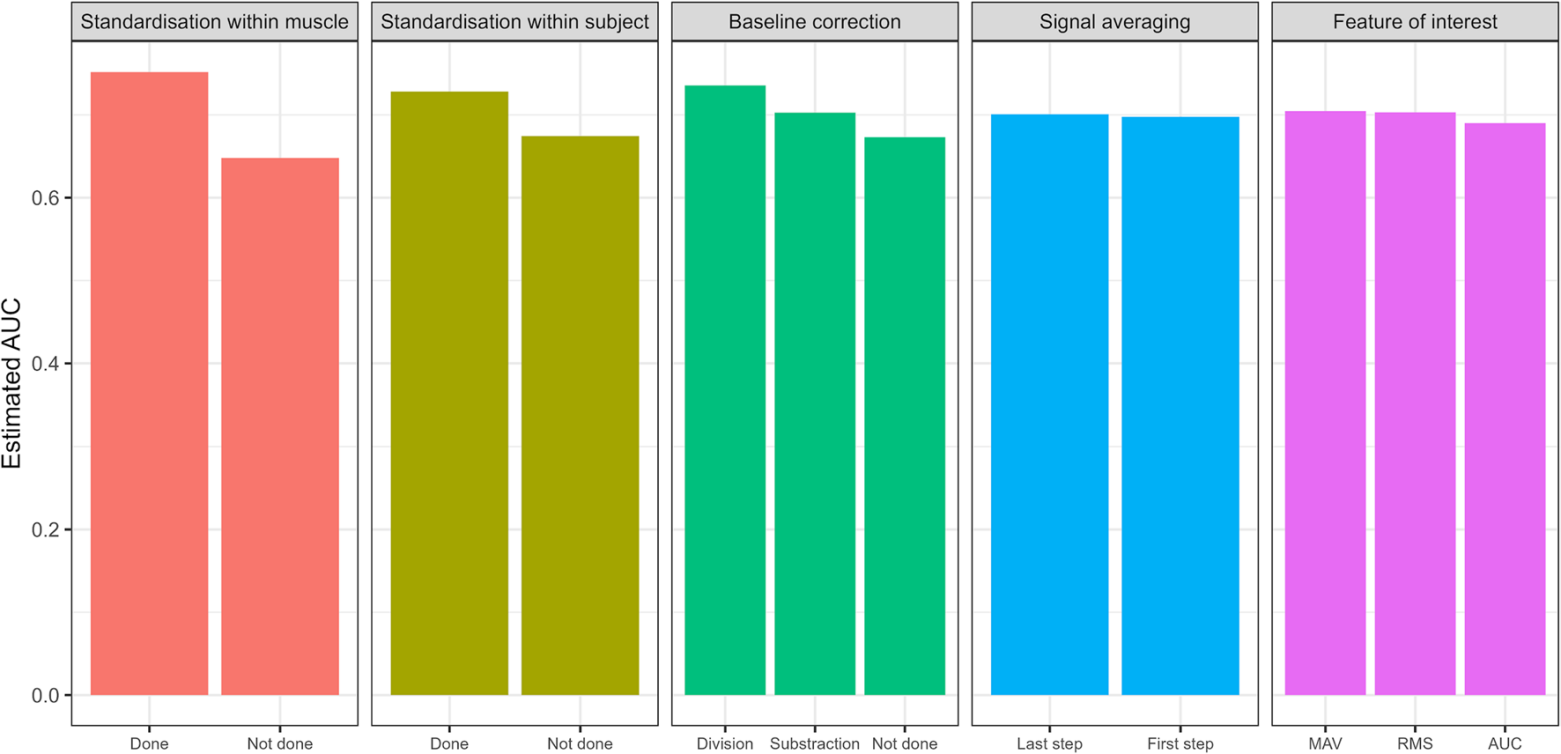
Partial dependence plot showing predicted pipeline AUC for each level of each variable in our random forest model. Higher expected AUC value indicates more positive impact on pipeline performance

The random forest model suggests that the standardisation within muscle was the most important, followed by the standardisation within subject and baseline correction. Signal averaging and the features of interest were classified as the least important. Please note that the random forest variable importance does not indicate which of the available options is the correct choice, such as which baseline correction is the best. This can be examined using the partial dependence plots for each variable in Fig. 5. Firstly, pipeline performance is improved when standardisation within muscles and subjects is conducted, compared to when it is not. Secondly, baseline correction by division shows increased predicted pipeline performance, compared to no baseline correction or baseline subtraction. Finally, different features of interest and signal averaging before or after other processing steps make little difference to predicted pipeline performance.

## Discussion

Although surface facial EMG is an established method for assessing emotional expressions, emotional cognition, and facial mimicry, there is no consensus on the optimal processing of the EMG signal. In fact, our literature review revealed that many different pipelines have been used to process EMG data. Thirteen of those pipelines directly corresponded to the pipelines assessed in this paper. Remarkably, according to our evaluation, the performance of these pipelines ranges from poor (AUC = 0.62) to very good (AUC = 0.89), showing a whole spectrum of sensitivity. The wide range in performance arises due to the lack of available guidelines for signal processing, and highlights the importance of and the need for more reliable research methods. A better understanding of the impact of different processing choices on the ability to detect emotional expressions is pivotal for future studies that will be able to analyse their data with the most sensitive pipeline recommended in this paper.

### Recommended processing practices

Based on the current outcomes, we recommend using the Aa_MAV_Bd_Ms_Sn pipeline (see *Naming the processing pipelines,* Fig. 3, Table 1 in Supplementary materials) to process the EMG signal when comparing facial muscle activation to detect even subtle emotional expressions. This pipeline had the best performance in the logistic model analysis, together with the Aa_MAV_Bd_Ms_Ss pipeline that differs only by the presence of within-subject standardisation (see Fig. 3). The Aa_MAV_Bd_Ms_Sn pipeline uses MAV, the mean absolute value, as a feature of interest extracted from the signal in each trial. It includes two processing steps that were recognised as most impactful on the performance: standardisation within muscle and baseline correction by dividing by baseline. In line with our findings, it has recently been shown that dividing the signal by baseline, instead of subtracting it, leads to a more reliable assessment of relationships between facial EMG responses to emotional stimuli and other behavioural indices of socio-cognitive processes (van Boxtel & van der Graaff, 2024). Conveniently, using the mean as a feature of interest might be more intuitive for the researchers new to the field, and easier for the broader scientific community to interpret, compared to using RMS or iEMG. This pipeline also averages the signal at the last processing step compared to the first, which is optimal when used in combination with its other processing choices (see Fig. 3b for the difference in performance between Aa_MAV_Bd_Ms_Sn and Ab_MAV_Bd_Ms_Sn, the pipeline with all the same steps except averaging the signal before the other processing steps). A step-by-step walkthrough of the recommended pipeline, together with the complete code from the processing script used in this paper, can be found in the Supplementary materials.

If, for specific reasons, like existing lab procedures, a preference exists for using a different pipeline, we nevertheless strongly recommend including both baseline correction and standardisation within muscle. All the pipelines that included those processing steps performed well (AUC > 0.75) and ranked in the 24 top-performing pipelines (see Fig. 3). However, it is worth pointing out that in studies involving EMG signals from multiple muscles involved in the expression of one emotion, the use of standardisation within muscle might obscure the contribution of individual muscles. In contrast, the experimental set-up used to collect our data involved recording each muscle contributing to one emotional expression only (*zygomaticus major* - happy, *corrugator supercilii* - sad), as is common practice in emotional facial mimicry research. Given that one includes baseline correction and standardisation within muscle in their processing of the EMG signal, other choices will likely have limited impact. Therefore, one can choose any feature of interest, to standardise data within subjects or not, and to average the signal before or after other processing steps based on their practical or theoretical relevance. If one’s processing pipeline does not include standardisation within muscle, standardisation within subjects can be included. The findings from this paper can be used flexibly by the researchers to make informed decisions about their specific data processing needs.

### Practical scope and applications

The findings of this paper are directly applicable to neuropsychological research on emotional expressions, emotional cognition, and facial mimicry that uses surface facial EMG. We aim to empower researchers to make informed decisions about their signal processing practices that will have a positive impact on their ability to extract relevant information from their EMG data. Importantly, we aim to make the optimal processing as accessible as possible, also to researchers with limited programming experience. To this end, we have made our data and annotated scripts, including all the different pipelines, available online (Rutkowska et al., 2023; https://github.com/TommasoGhilardi/EMG_Pipelines). This enables researchers to rerun all scripts on our data, and to adapt our scripts to run on their own data. In addition, our step-by-step walkthrough should allow them to recreate all processing steps in their respective software, even if they do not make use of the same underlying signal processing toolbox as used here. Thus, the analyses and material provided in this paper should enable researchers both to determine the best processing pipeline for their data and to implement it.

The ability to process surface EMG data in the most sensitive way to detect emotional expressions is especially important when the effect size is expected to be small or the statistical power to detect the effect is low, for example due to limits in the sample size. Both are widespread challenges in different fields of psychology and cognitive neuroscience (e.g., Szucs & Iodannidis, 2017; Lovakov & Agadullina, 2021) and pose problems because, in those instances, the effect of emotional stimuli could remain undetected due to the noise in the data and suboptimal processing. This is also particularly relevant to researchers collecting data from more challenging populations, such as infants or young children, which often results in only a few trials per participant (more noise) and smaller sample sizes than in research with adult participants. This kind of research might benefit the most from using our recommendations.

With the current paper, we aim to contribute to the open science movement, particularly to reproducibility, replicability, open methods, and pre-registrations, as follows. From the study conception to the publication, researchers in general make many choices (also called “researcher degrees of freedom”) that are often arbitrary from a methodological point of view or might even sometimes be aimed at achieving a statistically significant result (Wicherts et al., 2016). The latter is sometimes called “p-hacking” and increases the chance of finding a false positive result and inflating the effect sizes. This results in published research findings that are hard to reproduce on the same dataset or to replicate with a new one (Simmons et al., 2011; Ioannidis, 2005; Asendorpf et al., 2013). This paper specifically addresses one of these researcher degrees of freedom, namely data cleaning and processing. The processing of the data should be pre-specified prior to the start of the experiment, and should not be decided ad hoc by running the data through several processing pipelines and choosing the pipeline that provides the preferred results. Instead, the analysis pipeline can be documented as part of a pre-registration, along with the details about the study design before data collection. We encourage researchers to use our findings to decide on the EMG processing pipeline in advance and to include that in their pre-registration. We also encourage the researchers to use our published code to create and evaluate their own processing pipelines, and likewise share them together with the data at the time of publication.

To study other, non-emotion-related cognitive processes, our findings might be relevant to a limited extent. One example is the research on action prediction that measures the activity in the mylohyoid muscle with EMG (e.g., Cattaneo et al., 2007; Turati et al., 2013; Natale et al., 2014; Rutkowska et al., 2021). In the study presented here, we focused on predicting observed emotions from the interaction between the activities of two facial muscles. In contrast, the analysis of activity in the mylohyoid muscle relies on only one muscle located in the neck, which might decrease the importance of some of the standardisation measures in the preprocessing pipeline. In addition, the anatomical differences between small facial and larger neck muscles affect the recorded EMG signal, which may have an impact on the choice of appropriate processing methods (van Boxtel, 2001). Future research could address this by examining the optimal EMG processing practices in other fields of research, and this paper can provide the first stepping stone to these endeavours.

### Conclusions

So far, there has been no consensus on the best processing methods for EMG data in neuropsychological research on emotional expressions, emotional cognition, and facial mimicry. This paper took a data-driven approach to examine which processing practices are optimal for identifying emotional expressions in facial muscles. We found that three processing steps heighten the sensitivity of emotion effect on the EMG signal: baseline correction (preferably through division by baseline) and standardisation within muscles and within subjects. The choice of the feature of interest or the signal averaging before or after other processing steps had little influence. In addition to providing guidelines for designing new experiments, our recommendations can also be used for re-processing and re-analysis of existing data that might have been discarded due to null results arising from inadequate processing practices. We recommend the best-performing processing pipeline and provide a step-by-step walkthrough. This provides researchers with the knowledge to make informed data processing choices and with the tools necessary to implement it in their own research.

### Supplementary Information

Below is the link to the electronic supplementary material.

Supplementary file1 (PDF 253 KB)

## Data Availability

The data used in this study are available in a publicly available online repository (Rutkowska et al., 2023).

## Code availability

All the scripts used in the study, as well as the script for the recommended pipeline, are available in a publicly available online repository (Rutkowska et al., 2023) and on GitHub: https://github.com/TommasoGhilardi/EMG_Pipelines.
