# Supplementary material for: Optimal processing of surface facial EMG to identify emotional expressions: A data-driven approach
Source: Behav Res Methods. 2024 May 21;56(7):7331–44. doi: 10.3758/s13428-024-02421-4 (PMC11362446; doi:10.3758/s13428-024-02421-4)
Supplement: Supplementary file 1 — Supplementary file1 (PDF 253 KB) [file 13428_2024_2421_MOESM1_ESM.pdf]

Recommended processing pipeline: a step-by-step walkthrough

This document explains and provides the instructions for how to use the recommended pipeline for processing surface facial EMG data to detect subtle emotional expressions. This pipeline uses the data from Vacaru and colleagues' study (2021; Rutkowska et al., 2023) in BIDS format (Gorgolewski et al., 2016). The experiment involved 100 participants observing happy and sad facial displays whilst monitoring the activity of their *zygomaticus major* (*ZM*) and *corrugator supercilii* (*CS*) muscles to assess their facial mimicry. Facial mimicry is the mirroring of another person's facial expressions, although it is often smaller in magnitude than the mimicked expression. This pipeline processes the EMG data in an optimal way for increasing the sensitivity of the further analysis detecting sad and happy subtle emotional expressions from the activity of *ZM* and *CS* muscles, and their interaction. It is implemented using Fieldtrip (Oostenveld et al., 2011), an open-source toolbox for MATLAB.

This walkthrough also includes the preparation of data for processing and pre-processing. In data preparation, data-related paths are set and the event markers are mapped to the start of the presentation of emotional expressions on the screen (the start of the trial). In pre-processing, the two EMG channels from each muscle are re-referenced to each other. This is due to a special characteristic of the data we use, where the bipolar set up of the electrodes was recorded as unipolar using Brain Vision Recorder (Brain Products GmbH). The re-referencing leads to the data having one channel for each muscle, *ZM* and *CS*. Then, the loop for each subject is created. For each subject, the data is read in, filtered with a 20-500 Hz bandpass filter, and full-wave rectified. The artifacts are identified, the data is segmented into trials using the events, and the trials containing artifacts are rejected and removed from further processing. The pre-processed data is then ready for further processing.

This pipeline uses the pre-processed, but not averaged, data from each trial for all the processing steps. It first extracts mean absolute value (MAV) as the feature of interest, and then implements baseline correction by dividing the activity from each trial by the activity during the baseline period. Next, the data is standardised within muscles by z-scoring accordingly. Finally, the data is averaged across trials, which makes it ready for statistical analysis. The pipeline is provided as a separate script in the same repository as the data (Rutkowska et al., 2023) and in our github repository: https://github.com/TommasoGhilardi/EMG_Pipelines

Preparation

Before pre-processing, the data needs to be prepared in Fieldtrip. This entails setting the data-related paths, mapping the events in the data to the emotions they represent, and, in our case, re-referencing the channel because the electrodes were set up in a bipolar way, but the recording was done in a unipolar set up.

Setting data-related paths

In this section, we define the input and output paths for the data. The input path (bidsdir) is where the raw data is stored, while the output path (outputdir) is where the pipeline's processed output will be saved. This needs to be adjusted to user's own respective paths.

```
bidsdir   = 'C:\Users\tomma\Desktop\BabyBrain\Projects\EMG\Data\Bids'; % path of the data
outputdir = 'C:\Users\tomma\Desktop\BabyBrain\Projects\EMG\Data\Processing'; % path where to sa
```

Mapping the events

Here, we create a mapping between the response events and the emotions they represent (happy, neutral, and sad). This mapping will be used later in the pipeline to extract and analyze the corresponding EMG data. Each event signifies the start of the presentation of facial expressions to participants, and the start of the trial.

```
% Mapping events to emotions
response = [
     3     9
    12    18
    21    27
    30    36
    39    45
    48    54
    57    63
    66    72
    75    81
    84    90
    93    99
   102   108
   111   117
   120   126
   129   135
   138   144
   147   153
   156   162
   165   171];

happy   = response(:,1);
sad     = response(:,2);
```

Re-referencing the channels

The EMG data was recorded with a unipolar configuration, whilst the set up was bipolar, so there were two electrodes on each muscle site. In this section, we re-reference *ZM* and *CS* channels to each other, resulting in a bipolar channel configuration. This creates one channel per muscle.

```
% this is to combine two channels with a common reference into a singel bipolar channel
% this is done for corr and zyg

montage = [];
montage.labelold = {
  'corr1'
  'corr2'
  'zyg1'
  'zyg2'
  };    % old labels

montage.labelnew = {
  'corr'
  'zyg'
  };    % new labels
```

```
montage.tra = [
  1 -1 0 0
  0 0 1 -1
  ];    % channel matrix
```

Pre-processing

We want to process data from multiple subjects, so we initiate a for-loop to iterate through each subject. We also create an empty table called 'FinalData' to store the processed data for each subject as the loop progresses.

```
FinalData = table();

for subjindx = 1:100 % for each subject from number 1 to 100
```

Reading in and filtering

For each subject we will start by identifying the data and reading it. The ft_preprocessing function preprocesses the data as it is read. The data is filtered using a two-pass bandpass filter between 20 and 500 Hz, order 4.

```
    dataset = sprintf('%s/sub-P%04d/beh/sub-P%04d_task-observation_emg.vhdr', bidsdir, subjindx

    %% Reading and filtering data
    cfg = [];
    cfg.bpfilter  = 'yes';
    cfg.bpfreq    = [20 500]; % bandpass filter between 20 and 500
    cfg.bpfiltord = 4;
    cfg.bpfiltdir = 'twopass';
    cfg.dataset   = dataset;
    cfg.montage   = montage; % this was set in the previous section
    data = ft_preprocessing(cfg);
```

Rectifying the data

The data is full-wave rectified, a process where the negative values are converted to positive ones. In this case, we use the absolute values in the signal.

```
    data.trial{:} =  abs(data.trial{:}); % Rectification of signal
```

Reading in the events

In this part, we read in the events from the data. The events from the BrainVision system have a letter prefix, which we do not need, so we remove it to only keep the event number. We then filter out any events that don't correspond to the emotions of interest (happy or sad).

```
    % read events from data
    event = ft_read_event(dataset, 'type', 'Response', 'readbids', false);

    % removing letter from event value
    for i=1:numel(event)
        event(i).value = str2double(event(i).value(2:end));
    end
```

```
    % removing unnecessarey events
    event(~ismember([event.value], response)) = [];
```

Identify artifacts

In this section, we identify artifacts present in the data by performing the following steps:

- Segmenting the continuous data into 1-second segments with no overlap.
- Detecting artifacts by checking if the mean of each 1s segment is more than three standard deviations away from the mean of the overall channel. This is done independently for each channel.

```
    % Segmenting continuos data in 1s segments data with no overlap
    cfg = [];
    cfg.length  = 1;
    cfg.overlap = 0;
    data_seg    = ft_redefinetrial(cfg, data);

    % Mean and standard deviation rejection
    Me = mean(data.trial{:},2);     % mean for each channel
    Sd = std(data.trial{:},0,2);    % std for each channel

    M_seg       = cell2mat(cellfun(@(x) mean(x, 2), data_seg.trial,'UniformOutput', false));  %
    Rejection   = abs(M_seg(1,:)) > (Me(1)+3*Sd(1)) | abs(M_seg(2,:)) > (Me(2)+3*Sd(2));  % che
    % than 3sd
    Artifacts   = data_seg.sampleinfo(find(Rejection),:); % specific trials containing artifact

    % Print details
    Rej_N   = length(Rejection(Rejection == 1));
    Total_N = length(Rejection);
```

Segment into trials

In this part, we segment the data into trials using the event triggers that signal the presentation of the facial expressions and the start of each trial.

```
    %% Segment and clean data

    % Triggers selections
    numericvalue    = [happy, sad];
    stringvalue     = cell(size(numericvalue));
    for i=1:numel(numericvalue)
        stringvalue{i} = sprintf('R%3d', numericvalue(i));
    end

    % Segment into trials
    cfg = [];
    cfg.dataset                = dataset;
    cfg.trialdef.prestim       = 0.5;
    cfg.trialdef.poststim      = 2;
    cfg.trialdef.eventtype     = 'Response';
    cfg.trialdef.eventvalue = stringvalue;

    cfg         = ft_definetrial(cfg);
```

```
data_trl    = ft_redefinetrial(cfg, data);
```

Remove artifacts

We reject any trials containing artifacts we previously identified, resulting in clean EMG data for further analysis.

```
% Remove artifacts
cfg = [];
cfg.artfctdef.summary.artifact = Artifacts;
cfg.artfctdef.reject = 'complete';
data_trl_clean = ft_rejectartifact(cfg, data_trl);
```

Plotting the data

Here we can look the first 3 trials of the 1 subject after the preprocessing and artifact rejection

```
if subjindx ==1
    figure()

    subplot(2, 3, 1);
    plot(data_trl.time{1}, data_trl.trial{1}(1,:), 'b')
    title('Trial1')
    subplot(2, 3, 4);
    plot(data_trl.time{1}, data_trl.trial{1}(2,:), 'r')

    subplot(2, 3, 2);
    plot(data_trl.time{1}, data_trl.trial{2}(1,:), 'b')
    title('Trial2')
    subplot(2, 3, 5);
    plot(data_trl.time{1}, data_trl.trial{2}(2,:), 'r')

    subplot(2, 3, 3);
    plot(data_trl.time{1}, data_trl.trial{3}(1,:), 'b')
    title('Trial2')
    subplot(2, 3, 6);
    plot(data_trl.time{1}, data_trl.trial{3}(2,:), 'r')

    % Set labels for the two axes.
    ylabel(subplot(2, 3, 1), {'Corrugator','mV'});
    ylabel(subplot(2, 3, 4), {'Zygomaticus','mV'});

    % settign ylims
    ylim(subplot(2, 3, 1), [-1, inf]); ylim(subplot(2, 3, 2), [-1, inf]);
    ylim(subplot(2, 3, 3), [-1, inf]); ylim(subplot(2, 3, 4), [-1, inf])
    ylim(subplot(2, 3, 5), [-1, inf]); ylim(subplot(2, 3, 6), [-1, inf])

    xlim(subplot(2, 3, 1), [-0.7, inf]); xlim(subplot(2, 3, 2), [-0.7, inf]);
    xlim(subplot(2, 3, 3), [-0.7, inf]); xlim(subplot(2, 3, 4), [-0.7, inf])
    xlim(subplot(2, 3, 5), [-0.7, inf]); xlim(subplot(2, 3, 6), [-0.7, inf])

    % Set the title of the figure.
    sgtitle('EMG signal after basic preprocessing and artifact rejection');
```

```
end
```

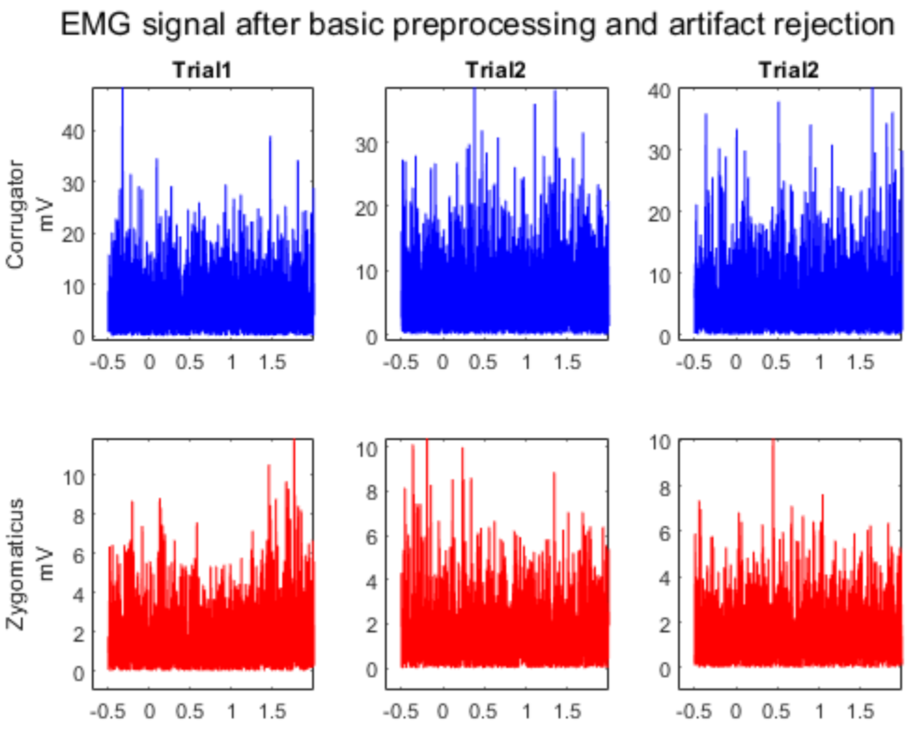

Processing

MAV and baseline extraction

In this section, we extract the mean absolute value (MAV) of each trial and the MAV of the corresponding baseline.

```
% Select baseline and data for each segment
for i=1:numel(data_trl_clean.trial)
    begsample = nearest(data_trl_clean.time{i}, 0);   % find sample closest to 0
    endsample = nearest(data_trl_clean.time{i}, inf); % find last sample (closest to inf)

    baseline_corr(i)  = mean(data_trl_clean.trial{i}(1,1:begsample-1));       % mav of the
    baseline_zyg(i)   = mean(data_trl_clean.trial{i}(2,1:begsample-1));       % mav of the
    active_corr(i)    = mean(data_trl_clean.trial{i}(1,begsample:endsample)); % mav of the
    active_zyg(i)     = mean(data_trl_clean.trial{i}(2,begsample:endsample)); % mav of the
end
```

Baseline correction

We normalise the data to the basline by dividing the MAV of each trial by the MAV of its respective baseline.

```
% Divide by baseline
Corr = (active_corr./baseline_corr)';
Zyg  = (active_zyg./baseline_zyg)';
```

Plot extracted fearures

Here again we take a look at the first 3 trials of the 1st subject after extacting the mean absolute value and baseline correction

```
muscles = categorical({'Corrugator', 'Zygomaticus'});

if subjindx == 1
    figure()

    subplot(1,3,1)
    hold on
    bar(muscles(1),Corr(1), 'b')
    bar(muscles(2),Zyg(1), 'r')
    title('Trial1')

    subplot(1,3,2)
    hold on
    bar(muscles(1),Corr(2), 'b')
    bar(muscles(2),Zyg(2), 'r')
    title('Trial2')

    subplot(1,3,3)
    hold on
    bar(muscles(1),Corr(3),'b')
    bar(muscles(2),Zyg(3), 'r')
    title('Trial3')

    ylabel(subplot(1, 3, 1), {'Mean absolute value'});
    sgtitle('EMG signal after MAV extraction and baseline correction');
end
```

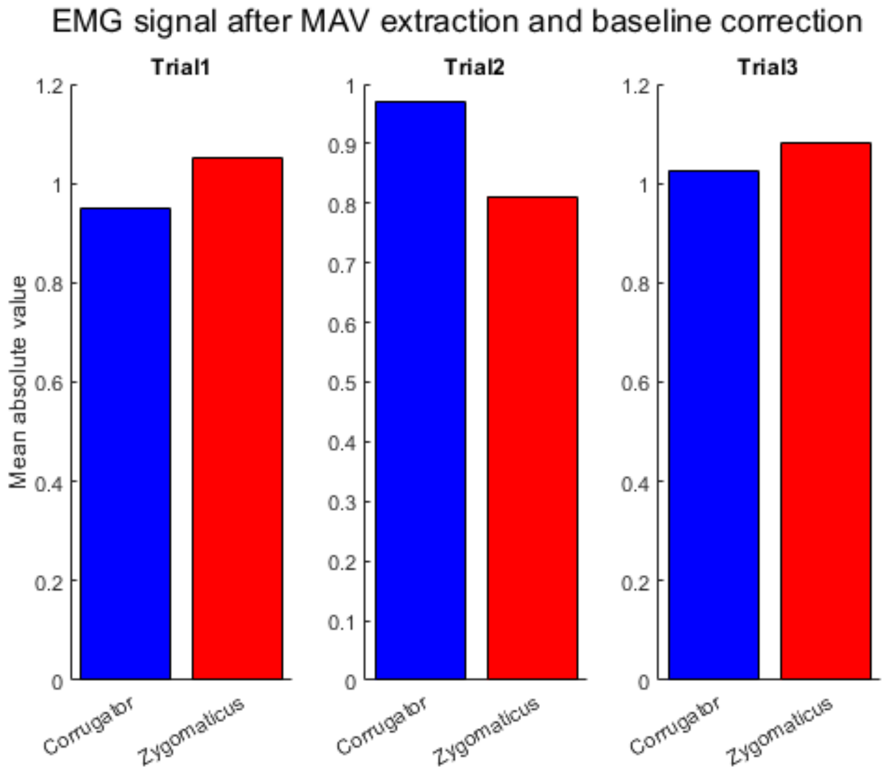

Trial division into conditions

We extract the condition of each trial based on the event triggers.

```
% map the trials onto condition codes
Condition = nan(size(data_trl_clean.trialinfo));
Condition(ismember(data_trl_clean.trialinfo, happy))    = 1;
Condition(ismember(data_trl_clean.trialinfo, sad))      = 2;
```

Integrating the data into a table

Here we put all the data from one subject into a table for standardisation.

```
% Integrate all in a table
Subject = repmat(subjindx,length(Condition),1);
MAV_corrected = table(Subject, Condition, Corr,Zyg);
```

Standardisation within muscle

The data is standardised within muscle using z-scoring, then saved into the table.

```
Standardization_WMuscle = zscore(MAV_corrected{:,3:4},0,1);         % standardisation of th
% within muscle

MAV_corrected{:,3:4} = Standardization_WMuscle;    % replacing data into the table
```

Plot standardized data

```
muscles = categorical({'Corrugator', 'Zygomaticus'});

if subjindx == 1
    figure()

    subplot(1,3,1)
    hold on
    bar(muscles(1),MAV_corrected.Corr(1), 'b')
    bar(muscles(2),MAV_corrected.Zyg(1), 'r')
    title('Trial1')

    subplot(1,3,2)
    hold on
    bar(muscles(1),MAV_corrected.Corr(2), 'b')
    bar(muscles(2),MAV_corrected.Zyg(2), 'r')
    title('Trial2')

    subplot(1,3,3)
    hold on
    bar(muscles(1),MAV_corrected.Corr(3),'b')
    bar(muscles(2),MAV_corrected.Zyg(3), 'r')
    title('Trial3')

    % Link the y-axes of the two subplots.
    linkaxes([subplot(1,3,1), subplot(1,3,2), subplot(1,3,3)], 'y');
    ylabel(subplot(1, 3, 1), {'Standardized mean absolute value'});
    sgtitle('MAV values after standardization');
end
```

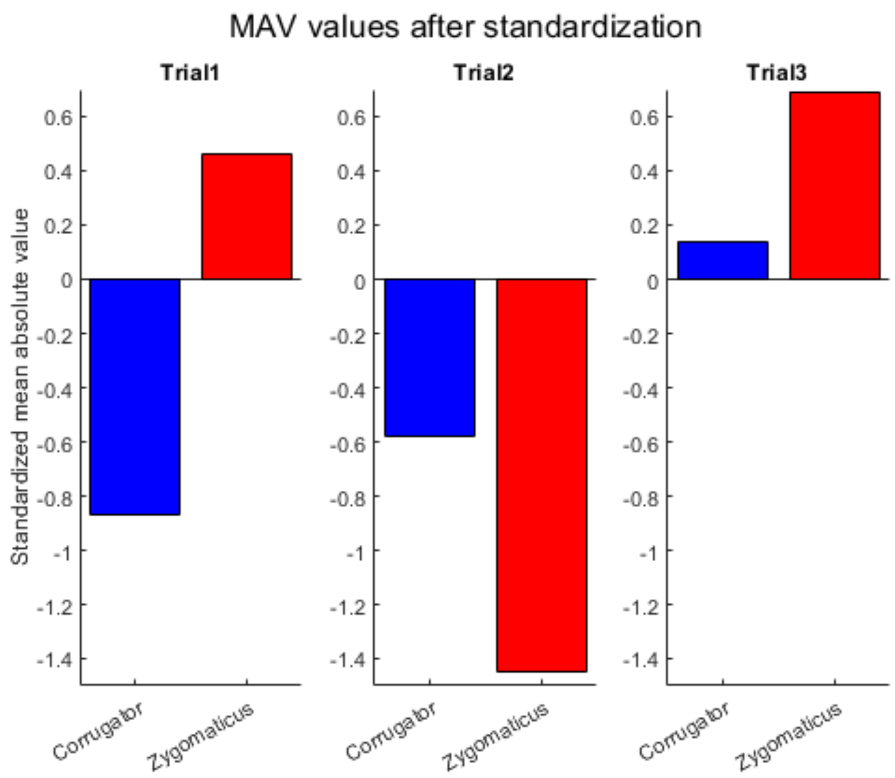

Appending the data

In this section, we append the data extracted for each specific subject to the general table called 'FinalData', which we created at the beginning of the pipeline. This process enables us to save the processed data from all subjects into a single table.

Before appending the data, we first calculate the average value for each condition (happy and sad) by finding the grouping between conditions using findgroups.

Additionally, to keep the script clean and efficient, we clear some variables that are no longer needed in the subsequent iterations of the loop or the rest of the script.

```
    % Extracting the average for each condition
    [Grouping , Group_table]  = findgroups(MAV_corrected(:,1:2));     % finding the grouping be
    % conditions
    Group_Values = splitapply(@mean, [MAV_corrected.Corr, MAV_corrected.Zyg], Grouping);     %
    % group

    Group_table.Corr = Group_Values(:,1);      % append averaged corrugator to the table
    Group_table.Zyg  = Group_Values(:,2);      % append averaged zygomativus to the table


    % Append the adata
    FinalData = [FinalData; MAV_corrected ]; % appending the data to the final table

    clearvars -except FinalData montage response sad happy *dir *Group   % clearing some variab

end
```

Save the data

After completing the processing of the EMG data for all subjects, we can now save our processed data to a CSV file. The 'writetable' function is used to write the contents of the 'FinalData' table to a file named 'ProcessedData.csv' in the specified output directory. This will allow for easy access and statistical analysis of the data.

```
writetable(FinalData, fullfile(outputdir,'ProcessedData.csv'))
```

References

Gorgolewski, K.J., Auer, T., Calhoun, V.D., Craddock, R.C., Das, S., Duff, E.P., Flandin, G., Ghosh, S.S., Glatard, T., Halchenko, Y.O., Handwerker, D.A., Hanke, M., Keator, D., Li, X., Michael, Z., Maumet, C., Nichols, B.N., Nichols, T.E., Pellman, J., Poline, J.-B., Rokem, A., Schaefer, G., Sochat, V., Triplett, W., Turner, J.A., Varoquaux, G., Poldrack, R.A. (2016). The brain imaging data structure, a format for organizing and describing outputs of neuroimaging experiments. *Scientific Data, 3* (160044). https://doi.org/10.1038/sdata.2016.44.

Oostenveld, R., Fries, P., Maris, E., & Schoffelen, J.-M. (2010). FieldTrip: Open Source Software for Advanced Analysis of MEG, EEG, and Invasive Electrophysiological Data. *Computational Intelligence and Neuroscience*, *2011*, e156869. https://doi.org/10.1155/2011/156869

Rutkowska, J.M., Ghilardi, T., Vacaru, S.V., van Schaik J.E., Meyer M., Hunnius, S., & Oostenveld, R. (2023). Optimising the processing of surface facial EMG to detect emotional expressions: recommended pipeline. Version 1. Radboud University. (dataset). https://doi.org/10.34973/ew6p-x929

Vacaru, S. V., van Schaik, J. E., Spiess, L., & Hunnius, S. (2021). No evidence for modulation of facial mimicry by attachment tendencies in adulthood: An EMG investigation. *The Journal of Social Psychology*, 1–15. https://doi.org/10.1080/00224545.2021.1973946
